# Digital Health Opportunities to Improve Primary Health Care in the Context of COVID-19: Scoping Review

**DOI:** 10.2196/35380

**Published:** 2022-05-31

**Authors:** Cícera Renata Diniz Vieira Silva, Rayssa Horácio Lopes, Osvaldo de Goes Bay Jr, Claudia Santos Martiniano, Miguel Fuentealba-Torres, Ricardo Alexandre Arcêncio, Luís Velez Lapão, Sonia Dias, Severina Alice da Costa Uchoa

**Affiliations:** 1 Faculty of Health Sciences Federal University of Rio Grande do Norte Natal Brazil; 2 Department of Collective Health Federal University of Rio Grande do Norte Natal Brazil; 3 Faculty of Health Sciences, Trairi Federal University of Rio Grande do Norte Santa Cruz Brazil; 4 Department of Nursing State University of Paraíba Campina Grande Brazil; 5 Universidad de los Andes Santiago Chile; 6 Department of Maternal Infant Nursing and Public Health University of São Paulo Ribeirão Preto Brazil; 7 Instituto de Higiene e Medicina Tropical Comprehensive Health Research Center Universidade Nova de Lisboa Lisbon Portugal; 8 Unidade de Investigação e Desenvolvimento em Engenharia Mecanica e Industrial Universidade Nova de Lisboa Lisbon Portugal; 9 Escola Nacional de Saúde Pública Comprehensive Health Research Center Universidade Nova de Lisboa Lisboa Portugal

**Keywords:** digital health, telehealth, telemedicine, primary health care, quality of care, COVID-19, pandemic, science database, gray literature

## Abstract

**Background:**

The COVID-19 pandemic brought social, economic, and health impacts, requiring fast adaptation of health systems. Although information and communication technologies were essential for achieving this objective, the extent to which health systems incorporated this technology is unknown.

**Objective:**

The aim of this study was to map the use of digital health strategies in primary health care worldwide and their impact on quality of care during the COVID-19 pandemic.

**Methods:**

We performed a scoping review based on the Joanna Briggs Institute manual and guided by the PRISMA (Preferred Reporting Items for Systematic Reviews and Meta-analyses) Extension for Scoping Reviews. A systematic and comprehensive three-step search was performed in June and July 2021 in multidisciplinary health science databases and the gray literature. Data extraction and eligibility were performed by two authors independently and interpreted using thematic analysis.

**Results:**

A total of 44 studies were included and six thematic groups were identified: characterization and geographic distribution of studies; nomenclatures of digital strategies adopted; types of information and communication technologies; characteristics of digital strategies in primary health care; impacts on quality of care; and benefits, limitations, and challenges of digital strategies in primary health care. The impacts on organization of quality of care were investigated by the majority of studies, demonstrating the strengthening of (1) continuity of care; (2) economic, social, geographical, time, and cultural accessibility; (3) coordination of care; (4) access; (5) integrality of care; (6) optimization of appointment time; (7) and efficiency. Negative impacts were also observed in the same dimensions, such as reduced access to services and increased inequity and unequal use of services offered, digital exclusion of part of the population, lack of planning for defining the role of professionals, disarticulation of actions with real needs of the population, fragile articulation between remote and face-to-face modalities, and unpreparedness of professionals to meet demands using digital technologies.

**Conclusions:**

The results showed the positive and negative impacts of remote strategies on quality of care in primary care and the inability to take advantage of the potential of technologies. This may demonstrate differences in the organization of fast and urgent implementation of digital strategies in primary health care worldwide. Primary health care must strengthen its response capacity, expand the use of information and communication technologies, and manage challenges using scientific evidence since digital health is important and must be integrated into public service.

## Introduction

Quality in health care is a multidimensional concept related to how offered services increase the probability of desired health outcomes. Health care quality also permeates correct care at the right time and in a coordinated manner, responding to the needs and preferences of service users, and reducing damage and wasted resources through a continuous and dynamic process [1]. Quality of care approximates health services to the population and has three dimensions: technical (accuracy of actions and the way they are performed), interpersonal (social and psychological relationships between care providers and users), and organizational (conditions in which services are offered, including globalization and continuity of care, coverage, coordination of actions, access, and accessibility to services) [2-4].

The COVID-19 pandemic led to immediate and profound social, economic, and health impacts, and required fast adaptation of health systems focusing on quality. Health systems, particularly primary health care (PHC), were pushed to maintain care routines, which required changes to maintain access and continuous management of health problems. This was possible owing to the creativity and innovation of professionals and managers, who introduced or expanded the use of information and communication technology (ICT) in the critical initial phase of the pandemic, where lack of coordination has negatively influenced access to health care [5].

ICT use has digital health as a great exponent in remote care strategies. This term is historically addressed as telemedicine or telehealth, which refers to communication and interaction tools between health care professionals and patients that provide remote health services and care as alternative to face-to-face appointments [6-8].

The use of telephones to answer doubts of patients, videos or text messages through mobile apps, and social media are helpful strategies for expanding the scope of health care by enabling population access. ICT also reduces the distance between patients and health professionals (eg, rural areas lacking health professionals), and facilitates appointment scheduling and renewal of prescriptions, thereby changing the professional-patient relationship and expanding personal health management [6,7,9-11].

The COVID-19 pandemic became a catalyst for expanding ICT worldwide [12]. Although digital health was recommended by the World Health Organization (WHO) [13-15] to reduce geographic barriers, its use increased only during the pandemic to maintain or increase access to health care, fight the pandemic, minimize economic impacts, and enable continuity of remote care [16,17].

Technological evolution may accelerate health care and improve access in the context of public health preparedness and response to outbreaks and emergencies. Despite these advances, the pandemic was challenging for health systems, mainly due to the lack of integration of technologies [17,18]. Considering the relevance of the topic for health and the wide use of ICT in PHC during the pandemic, we sought to gather knowledge about the quality of PHC using digital technologies. Therefore, the aim of this study was to map the use of digital health strategies in PHC worldwide and their impact on quality of care in the context of the COVID-19 pandemic.

## Methods

### Design

This scoping review was performed based on the Joanna Briggs Institute (JBI) manual [19] and guided by PRISMA-ScR (Preferred Reporting Items for Systematic Reviews and Meta-analyses Extension for Scoping Reviews) guidelines [20]. We also followed the steps proposed by Arksey and O’Malley [21] and Levac et al [22]: formulation of research questions, identification of relevant studies, study selection, data extraction and coding, analysis and interpretation of results, and consultation with stakeholders.

### Ethics Approval

The study was approved by the research ethics committee of the Faculty of Health Sciences of Trairí, Federal University of Rio Grande do Norte (CAAE: 47473121.3.0000.5568), and direct participation of participants involved in the study occurred only during consultation with stakeholders. The methodology used was previously reported in a protocol [23]. The term “telemedicine” used in the protocol [23] was replaced by “digital health” in this scoping review since it was considered to be more appropriate to reflect the broad scope of the study.

### Formulation of Research Questions

Study questions were defined by consensus among the authors and were formulated using the PCC (Population, Concept, and Context) mnemonic and the respective results of interest [19]: (1) Which countries used digital health in PHC in response to the COVID-19 pandemic? (2) What options of ICT were used in PHC in the context of the COVID-19 pandemic? (3) What is the impact of digital health on quality of health care delivery in PHC in the context of the COVID-19 pandemic?

### Identification of Relevant Studies

The following multidisciplinary health science databases were searched for relevant articles: MEDLINE/PubMed, Scopus, Web of Science, CINAHL, Embase, and LILACS. For gray literature, we consulted Google Scholar, WHO Global Research on Coronavirus Disease, PAHO Technical Documents and Research Evidence on COVID-19, Cochrane Library, medRxiv, SciELO Preprints, preprints.org, Open Grey, and Grey Literature Report.

The following types of studies and documents that addressed the research questions, focused on the use of remote strategies in PHC during the COVID-19 pandemic, and were available in full text were included: primary studies with quantitative, qualitative, or a mixed approach; experience reports; case reports; intervention studies; preprints; guidelines; manuals; reports; and government documents. No date or language filters were applied. Duplicate studies, protocols, literature reviews, opinion letters, and editorials were excluded.

### Study Selection

The search was performed between June 14 and July 14, 2021, using a three-step search strategy [24]: (1) exploratory search in two databases to identify descriptors and keywords, followed by construction of the search strategy, which was improved by a librarian using the Extraction, Conversion, Combination, Construction, and Use model [25]; (2) definition and search in all databases; and (3) manual search for additional sources in references of selected studies. The detailed search strategies are presented in Multimedia Appendix 1.

Study selection followed the PRISMA steps [26]: identification, screening, eligibility, and inclusion. A pilot study was independently conducted by two authors (CRDVS and RHL) using Rayyan software [27] to verify blinding, exclusion of duplicates, and selection of studies by titles and abstracts. Subsequently, full texts and reference lists of included studies were analyzed. For studies that did not meet inclusion criteria, a third author (SACU) was consulted.

### Data Extraction and Coding

Data extraction and coding ensured the consistency and reliability of results. Two authors (CRDVS and RHL) independently extracted all relevant data using an extraction form based on the JBI template [24], which was adapted by the authors, containing the following information: characterization of studies (first author, year, journal, country, type of study, participants); names of digital strategies adopted; types of ICT; characteristics of digital strategies in PHC; impacts on quality of care; and benefits, limitations, and challenges of digital strategies in PHC.

The database was organized in a Microsoft Excel 2016 spreadsheet and is provided for consultation in Multimedia Appendix 2.

### Analysis and Interpretation of Results

Data were analyzed qualitatively (narrative analysis) and quantitatively (absolute and relative frequencies). Thematic analysis [28] was structured based on familiarization with data, generation of initial codes, search for topics, review of topics, definition and naming of topics, and implications of studies. Results and narrative analyses are reported in tables and figures.

### Consultation With Stakeholders

Results of this review were presented to five stakeholders (ie, researchers with experience in digital health, ICT in health, and PHC) to fulfill the following objectives recommended by Levac et al [22]: preliminary sharing of study findings, considered a mechanism for transferring and exchanging knowledge, and development of effective dissemination strategies and ideas for future studies. The form questions are provided in Multimedia Appendix 3.

## Results

### Included Studies

**Figure 1:**
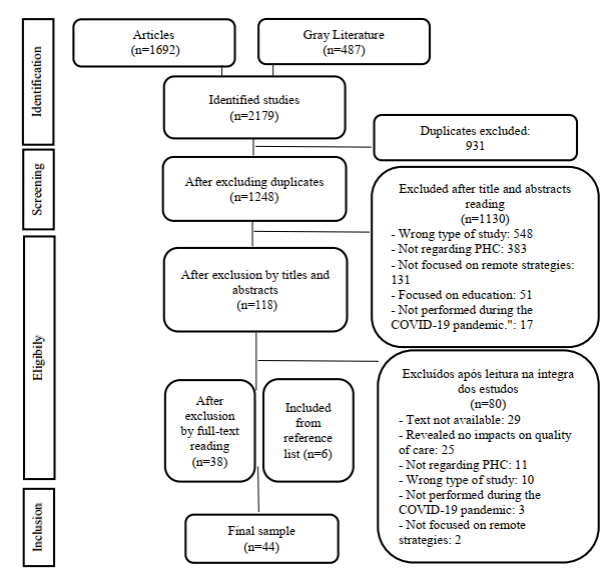
Flowchart of study selection for scoping review adapted from the Preferred Reporting Items for Systematic Review and Meta-Analyses (PRISMA).

A total of 2179 publications were identified (1692 peer-reviewed articles and 487 gray literature documents). After excluding duplicates, analysis of titles and abstracts, and full-text reading, 38 studies were included. The manual search of reference lists added 6 studies, resulting in a total of 44 publications for analysis (Figure 1). All included studies demonstrated the impacts of remote strategies on quality of care in PHC in the context of COVID-19.

### Characterization and Geographic Distribution of Studies

The studies included were mostly published during 2021 (28/44, 64%). Among the 44 articles, 27 (61%) used a cross-sectional design, 6 (14%) used qualitative investigation, 6 (14%) used mixed methods, 2 (5%) were cohort studies, 1 (2%) was an experience report, 1 (2%) was a case report, and 1 (2%) was an intervention study. The sample consisted mainly of patients (n=19, 43%), health professionals (n=13, 30%), medical or consultation records (n=9, 21%), and documented interviews with patients or health professionals (n=3, 7%).

**Figure 2:**
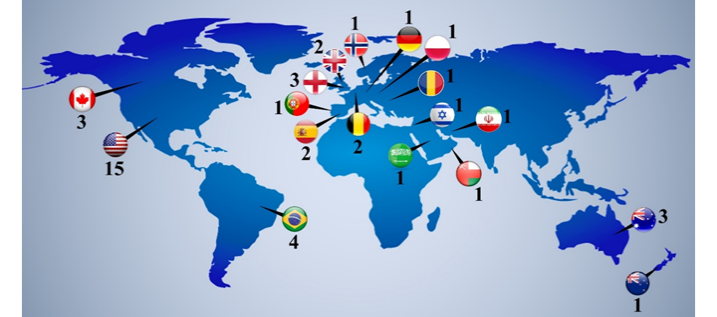
Distribution of countries that used digital strategies in primary health care. Numbers represent the number of studies performed in each country.

The studies covered 18 countries that used digital strategies in PHC; 18 studies were performed in North America (United States [29-43] and Canada [44-46]), 4 studies were performed in South America (Brazil [47-50]), 14 studies were performed in Europe (England [51-53], United Kingdom [54,55], Spain [56,57], Belgium [58,59], Norway [60], Portugal [61], Romania [62], Germany [63], and Poland [64]), four studies were performed in Asia (Israel [65], Oman [66], Saudi Arabia [67], and Iran [68]), and four studies were performed in Oceania (Australia [69-71] and New Zealand [72]). The characteristics of studies and distribution of countries that used digital strategies in PHC are described in Table 1 and Figure 2, respectively.

**Table 1:** Characteristics of the included studies.

Reference	Source	Country	Study design	Participants/sample
Alexander et al [29]	JAMA Network Open	United States	Cross-sectional	National audit of consultations (n=117.9 million)
Schweiberger et al [30]	Journal of Medical Internet Research	United States	Cross-sectional	Electronic medical records (n=45) and physicians (n=121)
Olayiwola et al [31]	JMIR Public Health Surveillance	United States	Cross-sectional	Consultation records (n=3617)
Atherly et al [32]	JMIR Public Health Surveillance	United States	Cross-sectional	Patients (n=1694)
Judson et al [33]	Journal of the American Medical Informatics Association	United States	Cross-sectional	Consultation records (n=1129)
Mills et al [34]	Journal of the American Health Association	United States	Cross-sectional	Patients (n=587)
Tarn et al [35]	Journal of the American Board of Family Medicine	United States	Cross-sectional	Medical records (n=202)
Adepoju et al [36]	Journal of Health Care for the Poor and Underserved	United States	Cross-sectional	Health workers (n=1344)
Ritchie et al [37]	Journal of the American Medical Directors Association	United States	Mixed methods	Health workers (n=79)
Drerup et al [38]	Telemedicine Journal and e-Health	United States	Cross-sectional	Patients (n=65)
Kalicki et al [39]	Journal of the American Geriatrics Society	United States	Cross-sectional	Medical records (n=873)
Chang et al [40]	Milbank Quarterly	United States	Cross-sectional	Health workers (n=918)
Thies et al [41]	Journal of Primary Care & Community Health	United States	Cross-sectional	Health workers (n=655)
Godfrey et al [42]	Contraception	United States	Cross-sectional	Medical records (n=534)
Juarez-Reyes et al [43]	Therapeutic Advances in Chronic Disease	United States	Qualitative investigation	Patients (n=6)
Bui et al [44]	Hamilton Family Health Team	Canada	Cross-sectional	Clinicians (n=126) and nurses (n=6)
Mohammed et al [45]	PLoS One	Canada	Cross-sectional	Clinicians (n=163) and nurses (n=37)
Donnelly et al [46]	BMC Family Practice	Canada	Mixed methods	Health workers (n=473)
Castro et al [47]	Revista Brasileira de Medicina da Família e da Comunidade	Brazil	Cross-sectional	Consultation records (n=329)
Dimer et al [48]	CoDAS	Brazil	Experience report	Consultation records (n=17)
Queiroz et al [49]	Acta Diabetologica	Brazil	Cohort	Patients (n=627)
Silva et al [50]	Ciência e Saúde Coletiva	Brazil	Cross-sectional	Clinicians and nurses (n=7054)
Sahni et al [51]	Cureus	England	Cross-sectional	Clinicians (n=312)
Leung et al [52]	BMJ Open Quality	England	Intervention study	Patients (n=12)
Tuijt et al [53]	British Journal of General Practice	England	Qualitative investigation	Patients (n=30) and caregivers (n=31)
Salisbury et al [54]	Journal of Medical Internet Research	United Kingdom	Mixed methods	Patients (n=1452) and health workers (n=12)
Murphy et al [55]	British Journal of General Practice	United Kingdom	Mixed methods	Medical records (n=350,966) and health workers (n=87)
Llamosas et al [56]	Physiotherapy	Spain	Case report	Patient (n=1)
Coronado-Vázquez et al [57]	Journal of Personalized Medicine	Spain	Cohort	Patients (n=166)
Morreel et al [58]	PLoS One	Belgium	Cross-sectional	Home visit records (n=15,655)
Verhoeven et al [59]	BMJ Open	Belgium	Qualitative investigation	Patients (n=132)
Johnsen et al [60]	Journal of Medical Internet Research	Norway	Cross-sectional	Clinicians (n=1237)
Lapão et al [61]	Journal of Medical Internet Research	Portugal	Mixed methods	Patients (n=35)
Florea et al [62]	International Journal of General Medicine	Romania	Cross-sectional	Clinicians (n=108)
Mueller et al [63]	JMIR Medical Informatics	Germany	Qualitative investigation	Patients (n=20)
Kludacz-Alessandri et al [64]	PLoS One	Poland	Cross-sectional	Patients (n=100)
Zeltzer et al [65]	National Bureau of Economic Research (NBER)/NBER Working Paper Series	Israel	Cross-sectional	Records from clinicians (n=4293) and patients (n=3.7 million)
Hasani et al [66]	Journal of Primary Care & Community Health	Oman	Qualitative investigation	Clinicians (n=22)
Alharbi et al [67]	Journal of Family and Community Medicine	Saudi Arabia	Cross-sectional	Patients (n=439)
Jannati et al [68]	International Journal of Medical Informatics	Iran	Cross-sectional	Patients (n=400)
Isautier et al [69]	Journal of Medical Internet Research	Australia	Cross-sectional	Patients (n=596)
Javanparast et al [70]	BMC Family Practice	Australia	Qualitative investigation	Patients (n=30)
Ervin et al [71]	Australian Journal of Primary Health	Australia	Cross-sectional	Clinicians (n=24)
Imlach et al [72]	BMC Family Practice	New Zealand	Mixed methods	Patients (n=1010)

### Nomenclatures of Adopted Digital Strategies

**Figure 3:**
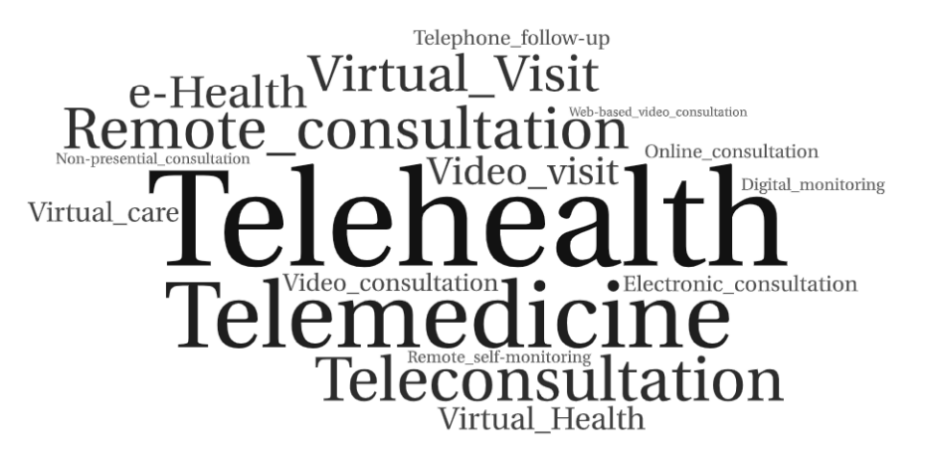
Word cloud with nomenclatures used to refer to digital strategies in primary health care.

Nomenclatures regarding remote care strategies varied considerably among studies, with the terms “telehealth” [30,33,36,44,45,47,54,55,60,63,64,67-69,72] and “telemedicine” [29,31,32,38,39,46,47,51,59,61,63,69,71] being the most frequent. The following terms were also mentioned: teleconsultation [40,58,71], virtual visit [41,48,58], virtual health/eHealth [35,51], remote consultation [37,50,56,65], electronic consultation [35,62], telephone follow-up [35,66], video visit [35,70], video consultation [34,49], online consultation [69], virtual care [53], web-based video consultation [69], digital monitoring [72], nonpresential consultation [52], and remote self-monitoring [43]. Figure 3 shows the word cloud representing the most commonly used nomenclatures.

### Types of ICT Employed

A total of 39 of the 44 studies (89%) mentioned the types of ICT used in PHC. Telephone calls had the highest number of records (29/39, 74%) [30,31,33-38,40,44-48, 50,52,53,55-60,62,65,66,69,70,72], followed by video calls (25/39, 64%) [30,31,33-41,43-48,55,60,62-65,69,72], patient portal (11/39, 28%) [31,33,35-37,40,42,44,58,61,72], smartphone apps (5/39, 13%) [44,49,52,54,68], text messages (3/39, 8%) [35,46,60], email (3/39, 8%) [46,62,72], electronic medical record (2/39, 5%) [31,72], and social networks (1/39, 3%) [46]. We highlight that many studies used more than one type of technology, mainly phone and video calls.

Moreover, the following electronic platforms and apps were used to conduct services: WhatsApp, Updox, Epic MyChart, Doximity, Facetime, Skype, Zoom, Telegram, iCARE-DATA, Babylon GP at Hand (BGPaH), EyerCloud, DRiQ, Aid Access, Telus PS Suite, eVisit Ontario Telemedicine Network, and Multimorbidity Management Health Information System (METHIS).

### Characteristics of Digital Strategies in PHC

**Figure 4:**
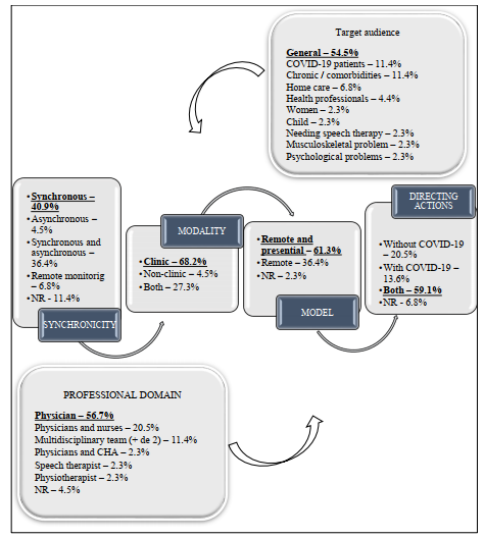
Characteristics of remote strategies in primary health care.

We analyzed the target audience, professionals involved, direction, synchronicity, and modality and model of actions in PHC (Figure 4).

Actions were mostly directed to the general public (ie, any health status or characteristic) [29,31,32,34-36,38,40-42, 44,46,48,49,51,53,55,56,58-60,63,67,71]. Regarding professionals who conducted the actions, the majority were clinicians [29-31, 34, 36-38, 42, 44-46, 49-51, 54, 55, 57, 58, 60, 63-66, 69, 71, 72] and nurses [35,39,41,43,47,48,56,70,72]. Actions were directed toward people with and without COVID-19 [31,32,34,36,38,40,42, 44,49,51,53-56,58-63, 65,67,68,70-72]. Synchronous interaction [31-33, 40,42,44-46,51,52,54,63-66,68,70,71] was the most frequently reported interaction type. The clinical modality was the most commonly reported [29-31, 33, 36-38, 40-46, 48-52, 54, 58-60, 63, 65-67, 69, 71, 72], referring to the following actions: consultations, renewal of medical prescriptions, exams, follow-up, health guidelines, issuance of certificates, treatments, screening, monitoring, diagnosis, management of chronic conditions, referrals, clinical self-monitoring, and risk classification. Remote consultation associated with in-person actions was the most prevalent model [29-32,34-38,40,42-46,49,51,53,54,56,57,60-64,67].

### Impacts on Quality of Care

Studies reported the impacts of remote strategies on technical, interpersonal, or organizational dimensions of quality of care. Positive impacts were highlighted in 19 of the 44 (43%) studies [31,33,34,38,41,42,45,47-50, 52,56,57,60,62,64,65,70], negative impacts were mentioned in 6 (14%) studies [36,40,51,53,63,69], and positive and negative impacts were mentioned in 19 (43%) studies [29, 30, 32, 35, 37, 39, 43, 44, 46, 54, 55, 58, 59, 61, 66-68, 71, 72].

Technical [29-31,33-40,42-44,46,47,49-69,71,72] and organizational [29-43,45-50,52-61,64,65,67-72] dimensions were the most cited, followed by the interpersonal dimension [31,35-43,46-48,52,53,55,59,64,65,67-70,72]. More than one dimension of quality of care was directly or indirectly addressed in most studies.

Textbox 1 summarizes the positive and negative impacts on dimensions of quality of care evidenced in the studies.

**Impacts on dimensions of quality of care.**

**Technical dimension**

*Positive impacts*

- Security in care provision [31,33,35,37,38,42,43,46,49,52,55,57-59,61,64,66,67,71,72]
- Technical quality of information and communication technology [31,34,49,52,57,60,62,68]
- Technical accuracy [42,44,47,49,52,58,67]
- Resolvability [33,35,43,55,64,72]
- Support for clinical decision-making [50,65]
- Reliability [64,68]
- Utility [30]
- Attendance [56]
- Privacy [31]

*Negative impacts*

- Technical inaccuracy/inaccuracy [36,37,46,53,55,59,61,69,72]
- Low quality of consultations [33,36,51,63,69]
- Lack of assessment of vital signs and physical exams [29,59,63,69]
- Selective resolvability [39,40,53,54]
- Insecurity of data privacy [36,54,61,67]
- Discrepancy between professional conduct [66]

**Interpersonal dimension**

*Positive impacts*

- Trust and bond with professionals improved adherence [43,47,48,65,67,68,70,72]
- Ease loneliness [39,46,52,59,64,70,72]
- Professional respect [31,42,43,64,68]
- Active listening [38,47,59]
- Positive interpersonal communication [38,41,64]
- Humanization of care [31]

*Negative impacts*

- Loss of nonverbal communication; lack of eye contact or touch [36,43,53,55,59,68,69,72]
- Interpersonal communication hampered by technology, speed of consultation, or memory difficulties of patients [39,40,53,59,69]
- Great emotional burden and stress [40,55]
- Fear of not being resolutive compared with face-to-face modality; insecurity [35,37]

**Organizational domain**

*Positive impacts*

- Continuous care [32-34,38,39,42,43,47,48,54-57,59,60,65,67,68,70]
- Economic, social, geographical, time, and cultural accessibility [29,31,34,38,42,52,54,55,57,67,70-72]
- Coordination of care [31,33,34,47,49,50,52,57-59,64]
- Access [42,43,45-47,54,58,65,72]
- Integrality of care [31,38,42,57,67,71]
- Optimization of consultation time [15,38,43,52,55,61,64]
- Economic efficiency [33,38,52,57,64]
- Organization of the work process [31,41,64]
- Increased demand for assistance [38,64,72]
- Planning of quality improvement [31,41]
- User-friendly technologies [33]
- Community engagement [37]

*Negative impacts*

- Reduced access; evidence of inequity [32,35-37,39,40,54,55,59,69]
- Reduced integrality of care [36,37,40,46,53,54,59,69]
- Digital exclusion [35-37,39,40,54,68,69]
- Lack of planning in defining the role of the team; disarticulation between actions and needs of the population [37,46,53,55,59,69,71]
- Reduced training of professionals using information and communication technology [37,53,54,59,61,69]
- Reduced continuity of care [30,36,40,54,58]
- Reduced coordination of care; fragile remote-presential articulation [37,46,54,55,68]
- Lack of professionals; high turnover [37,54,59]
- Reduced accessibility [30,32,53]
- Lack of support in internet technologies [36,69,71]
- Reduced active search in the community [53]

### Benefits, Limitations, and Challenges of Digital Strategies in PHC

The following benefits of digital strategies in PHC were highlighted in the studies: (1) acceptability and patient satisfaction [29,31,34,36,38,43-45,47,52,54,56-58,62-64,67,68]; (2) great possibility of sustainability in the postpandemic period [31,32,38,40,43,54,55,58,62,64,68-72]; (3) increased frequency of people seeking care in PHC, especially in remote areas with difficult access and little face-to-face demand [29,34-36,41,46,49,50,53, 55,59,70-72]; (4) great safety against COVID-19 transmission [33,35,38,42,47,54, 55,57-59,61,66,71,72]; (5) time and cost savings due to geographic displacements [33,34,36,38, 43,49,55,63,68,70,72]; (6) organization of work process and scheduling of face-to-face and remote demands [31,39,41,47,48,53,54,63]; (7) faster service [33,52-54,61,66-68]; (8) reduced need for referrals to secondary care and hospitalizations [33,35,44,50,52,57,65]; (9) great comfort and practicality [34,36,42,43,68,72]; (10) optimization of training, meetings, and education of professionals [31,33,41,49,59]; (11) opportunity to be present in patients’ lives, which benefits emotional health [30,32,43,44]; (12) fast home screening in cases of clinical changes [31,44,57]; (13) better communication with patients [46,64]; (14) great facility of use of technological tools and opportunity to overcome technological limitations [52,64]; (15) advantage of video calls over other tools [39,63]; (16) possibility of choosing the attendance modality [54]; (17) anonymity in situations that generate stigma, such as abortion care [42]; and (18) increased possibility of contacting inaccessible patients [61].

Conversely, the following limitations and challenges of digital strategies in PHC were identified: (1) difficulty in accessing internet, poor connectivity, digital divide (ie, more people with access to telephones and less to video calls) or digital desert (ie, people without access to technologies) [32-36,38-40,42,45-47,54,55,59,64,67,68,72]; (2) increased need for training professionals and the population regarding digital health [36,37,39-41,44-47,50,51,55,59,61,66,69-71]; (3) great diagnostic imprecision and professional misconduct due to absence of physical examinations [39,43,52, 54-56,59-61,65,68,69,72]; (4) inconsistent platforms, with errors in data storage, limited resources, or both [31,33,38,41,43,45,58,60,64,68,71]; (5) difficult communication with the elderly, children, and people with disabilities or dementia [37-39,46,48,53,55,59,69]; (6) lack of planning regarding management of services [40,41,46,52,54,55,61,71]; (7) uncertainty about privacy and confidentiality of personal data [35,36,41,61,63,66,67]; (8) rapid implementation of remote services without prior guarantee of equitable access [30,42,55,63,71,72]; (9) poor support from information technology professionals [31,36,41,43,66,71]; (10) great need for good articulation between remote and face-to-face modalities to meet demands [39,40,60,63,70]; (11) mental stress in health workers [37,43,46,55,59]; (12) lack of health professionals, high turnover of professionals, or both [37,54,57,59,67]; (13) possible increase of chronic conditions (eg, certain groups of people who stopped seeking services) and side effects due to excessive self-medication [53,55,58,59]; (14) telephone calls are used but not resolutive [34,35,53,64]; (15) low acceptability of professionals toward new remote workflows [46,51,55]; (16) difficult clinical monitoring of patients at home [51,57,64]; (17) difficulty regarding early identification of more complex health demands [31,59,69]; (18) delayed administrative tasks of health teams due to increased care demands [47,59]; (19) fast and urgent care [53,54]; (20) difficult articulation between professionals to meet more complex demands [44,54]; (21) difficulty regarding referral to other services [46]; (22) poor resolution in situations of risk at home (ie, domestic violence) [72]; (23) reduced supply of services [32]; and (24) difficulty in long-term follow-up of patients [49].

## Discussion

### Main Findings and Relation to Existing Literature

This scoping review demonstrated that the COVID-19 pandemic impacted health care in PHC worldwide (ie, fast implementation or increased use of remote care strategies or both) to mitigate the pandemic and ensure continuity of activities [73]. Various terms to refer to remote strategies were found in the literature [8,74]. Beyond concepts, technologies and tools are important components for health care systems, supporting the interaction among health care professionals or between health care professionals and patients [9]. The WHO [13,14] suggests telemedicine or telehealth to define distance care using ICT, whose purpose is to provide health care services in situations where distance or geographic barriers hinder the provision of care. Recently, “digital health” was introduced as an umbrella term, covering the use of electronic and mobile technologies (eg, advanced computer science, artificial intelligence, and big data) to support health and emerging health care areas [75].

The WHO and others [75,76] highlight the importance of digital technologies for achieving sustainable development goals and the advance of universal health coverage as opportunities to face challenges of health systems (ie, delayed provision of care, and reduced demand, adherence, and geographic accessibility) and increase coverage, accessibility, and quality of actions.

Telephone and video consultations are efficient tools for offering digital health [77,78]. Although telephone may increase follow-up contact and is more accessible than tools that require an internet connection, the assessment of severity and health status is compromised due to the absence of eye contact [79].

Telephone and audio consultations were recognized as telehealth modalities during the COVID-19 pandemic to support social distancing [80]. Although video consultations were rare in many locations before the pandemic [77], they are superior to phone calls, mainly due to eye contact and better communication for building bonds. Nevertheless, technical problems are more frequent when using digital strategies, and people need a stable connection to the internet, which may raise questions about the relationship between equity and the type of technology used [81-84]. For greater benefits, the literature indicates that the use of technology should be simple, consistent with local workflows, convenient for users, offer advantages over face-to-face consultations [76,85,86], and complement other existing technologies.

Results of this study corroborate with those of Breton et al [87], in which phone calls and video calls were identified as the most frequently used remote technologies, especially in the first months of the pandemic. We highlight that communication between health services users and professionals, mainly regarding platforms that ensure safety and reliability in the context of health care [88], is an important measure to be adopted due to the increased offer of newly developed applications.

The results of this scoping review also revealed the positive and negative impacts of remote strategies on quality of care in PHC worldwide, suggesting different types of organization (eg, fast or urgent implementation) of digital strategies. Safe offer of care, technical quality and accuracy, and resolvability were the positive impacts most frequently reported in the technical dimension. By contrast, technical inaccuracy or imprecision, consultations with poor quality, lack of detailed physical examination, and selective solving of problems were also observed.

The interpersonal dimension was characterized by trust and bond with professionals that facilitated adherence to technologies, increased the possibility of talking to someone, alleviated loneliness caused by isolation, and improved respect between professionals and patients. From another perspective, we also found loss of nonverbal communication, lack of physical contact, difficult communication aggravated by technologies, and negative and stressful emotional load among professionals as negative impacts.

The impacts in the organizational dimension were the most frequently identified in the included studies, which strengthened continuity of care; economic, social, geographical, time, and cultural accessibility; coordination of care; access; integrality of care; and optimization of appointment time and efficiency. Negative impacts were also observed in this dimension, such as reduced access to services, inequity, and unequal use of services offered; digital exclusion of part of the population due to lack of technologies, connectivity, or knowledge regarding use; reduced integrality of care; lack of planning for defining the role of professionals; disarticulation of actions with real needs of the population; impaired continuity of care; reduced coordination of care; fragile articulation between remote and face-to-face modalities; and unpreparedness of professionals to meet demands mediated by ICT.

One study [89] that verified how the pandemic impacted primary care services suggested digital health as an inflection point for PHC and the only alternative for restructuring the workflow of health care providers during the pandemic. The latter may have also contributed to the impaired quality of health care, especially for the elderly and people with preexisting health conditions (ie, psychological problems, addictions, or victims of domestic violence).

Issues limiting technological barriers and ethics in the use of information might be linked to work organization, health financing, and lack of familiarity of professionals and patients [6]. When properly available, patients considered digital health to be satisfactory and safe, and felt comfortable when trusting relationships with professionals and person-centered practices were present.

In PHC, preexisting virtual solutions to COVID-19 served as opportunities to support public health responses in combating the pandemic and minimizing the risk of exposure [90-93]. The adaptation of health systems based on PHC and training of professionals regarding the use of digital tools to fulfill clinical responsibilities, which previously required face-to-face contact, were also useful [90]. Studies also highlighted the relevance of digital strategies in preventive and health promotion actions, such as remote monitoring of clinical signs; management of chronic diseases and medication; and guidance on healthy lifestyle, exercises, and eating habits [94,95].

Studies conducted before the COVID-19 pandemic demonstrated the importance of digital health in expanding access in PHC [82,96,97], even though face-to-face care was preferred [98]. Positive experiences were associated with planning according to the health needs of the population [99-101], whereas health professionals complained about insufficient remuneration, unavailability of technologies, and lack of standardization [102,103]. Based on these prepandemic experiences, digital strategies in PHC were an option to mitigate barriers and increase access for hard-to-reach populations. During periods of greater restriction and social isolation due to the COVID-19 pandemic, the reality of virtual assistance was extrapolated beyond populations with difficulties in accessing services. This fact allowed us to observe different results regarding the strengthening of digital health or predominance of persistent problems that depended on decision-making factors of governance to provide broad coverage of technologies (complementary or alternative) to populations. In fact, in most situations, digital health was adopted without the support of a national strategy.

The results of this study emphasize the benefits, limitations, and challenges of remote strategies in PHC, offering lessons during a global public health crisis. In this sense, quality of care in PHC can still be improved with consolidation and advances in digital health.

### Implications for Practice and Research

According to the Pan American Health Organization [104], ICTs are essential to increase access of citizens to high-quality PHC, regardless of their distance from large urban centers. Technologies are becoming the primary method in which people, governments, and health institutions work, communicate, and generate and exchange knowledge. In this context, we must reflect on how remote technologies and strategies can support and strengthen essential characteristics of PHC, since this is the first point of contact for people, and offers comprehensive, accessible, and community-based health care. PHC also offers health promotion and prevention, treatment of acute and infectious diseases, control of chronic diseases, palliative care, and rehabilitation to individuals, families, and communities [105].

This study demonstrates that the fast transition and expansion of digital health impacted access and quality of care in PHC worldwide, even considering that health needs, policies, management, and financing differ between countries. PHC must take advantage of the lessons learned from the COVID-19 pandemic, strengthen its response capacity, balance the offer of new modalities of care with expanded use of technologies, and be more equitable and accessible. In contrast, equity of health care supply is beyond the power of action of health professionals or management of local services, since it is a larger and structural problem that depends on the integrated actions and engagement of public and social policies.

PHC services must be aligned with the needs and satisfaction of the population, while efforts must be made to perform self-assessments and improve quality of in-person and remote processes. Planning and intersectoral articulation at the management level, along with investment in financial and human resources are essential to improve the cost-effectiveness of remote care. Furthermore, technical and operational infrastructure is imperative for using technologies, strengthening security and protection of the patients and professional data.

Services and actions exceeding needs increase costs and do not improve results regarding patient-centered care and needs [106,107]. Moreover, health outcomes are worse, and costs are high when care is not based on the needs of the population. For digital health strategies in PHC, Lillrank et al [108] recommend planning actions by homogeneous groups with similar health needs, and organizing the supply of care considering demand, severity, and duration of needs, according to demand and supply–based operating modes. This organization could also facilitate continuity of care and optimize the work process using remote strategies.

The identification of gaps in the literature is expected in scoping reviews. As the COVID-19 pandemic changed the provision of services at all levels of care worldwide (eg, expansion of remote care strategies), directions for future research are challenging because the long-term impacts are unknown. Based on the observations from this scoping review, we recommend the following primary studies focused on remote strategies in PHC, especially in countries that have not yet investigated the topics discussed here: (1) assess implementation and differences between health systems (either public and private or with different forms of management and financing) based on the principles of universality and universal coverage; (2) assess the effectiveness and safety of remote strategies between users, professionals, and health managers; (3) monitor the impacts of remote strategies on quality of care and investigate how to enhance quality; and (4) perform intervention studies to investigate innovative strategies or approaches to improve clinical practice. Moreover, systematic reviews with meta-analysis could be performed to (1) assess the impact of remote strategies on clinical outcomes in vulnerable populations and (2) follow-up of patients with COVID-19 complications using ICT.

### Consultation With Stakeholders

In the consultation stage, stakeholders were asked about ideas for future research, applicability of the results, and dissemination strategies. From the perspective of the participants, this scoping review can stimulate development agencies to finance ICT in PHC; reflect on cost-effectiveness of digital health to achieve greater adherence to therapeutic plans, reduce disease transmission, and prevent injuries; demonstrate the benefits of using digital health for monitoring indicators, goals, and indices in PHC, and for health surveillance; and support health professionals with lessons learned for improving care in remote mode.

Regarding the possibilities of disseminating the results, the following suggestions were discussed: scientific dissemination (indexed journals, conferences, and workshops); disclosure by health secretariats; creation of networks with interested social agents; linking of agents to research groups to approximate academia from health services and the general population; meetings and debates with local and national health managers; and adaptation of dissemination of results according to the local culture, choosing the most accessible means of communication (ie, social networks).

When asked about ideas for future research, the following were suggested: action research with health managers and professionals focusing on solutions for digital inclusion of vulnerable populations; sectorial studies inserted in PHC (eg, sectional and intervention research designs regarding digital pharmaceutical and oral health care industries); studies investigating the acceptability of remote strategies by specific groups and its associated factors (eg, age, gender, socioeconomic status, preexisting health conditions, and beliefs); and long-term follow-up of patients using remote monitoring in PHC.

### Strengths and Limitations

This scoping review is the first to broadly map evidence regarding the use of remote strategies in PHC and its impacts on quality of care in the context of COVID-19. The study met the criteria for scoping reviews [24,109], and followed methodological references, checklists, and published protocols [23].

We did not conduct a meta-analysis [23] or assess the quality of studies. However, these steps are not essential due to the exploratory and descriptive nature of a scoping review. The search was performed to retrieve the highest number of publications regarding the topic, rather than focusing on studies with the highest standards of scientific rigor. Even though databases for peer-reviewed publications and gray literature were included with no filter limits and a high-sensitivity search strategy was performed, we do not know to what extent relevant studies and important databases were included.

### Conclusion

This review provides information on the use of digital strategies in PHC and its impacts on quality of care during the COVID-19 pandemic. Confronting a public health situation of such proportion sheds light on realities that were not as evident previously. Given the importance of digital health in the current global health situation and the possibility of integrating and advancing this strategy after the pandemic, primary care must strengthen its response capacity, expand ICT use, and manage challenges using scientific evidence.

The number of digital health initiatives launched worldwide without a scientific basis during the pandemic had its foundation in the health crisis. Digital health needs to be improved and expanded to strengthen primary care and health systems.

## Appendix

Multimedia Appendix 1 Search strategy.

Multimedia Appendix 2 Data extraction spreadsheet (original language).

Multimedia Appendix 3 Stakeholders consultation guide.

## Abbreviations

ICT: information and communication technology
JBI: Joanna Briggs Institute
PCC: Population, Concept, and Context
PHC: primary health care
PRISMA: Preferred Reporting Items for Systematic Reviews and Meta-analyses
PRISMA-ScR: Preferred Reporting Items for Systematic Reviews and Meta-analyses Extension for Scoping Reviews
WHO: World Health Organization
