# Supplementary material for: Digital Health Opportunities to Improve Primary Health Care in the Context of COVID-19: Scoping Review
Source: JMIR Hum Factors. 2022 May 31;9(2):e35380. doi: 10.2196/35380 (PMC9159467; doi:10.2196/35380)
Supplement: Multimedia Appendix 1 [file humanfactors_v9i2e35380_app1.doc]

| **Multimedia Appendix 1. Search Strategy** | |
| --- | --- |
| POPULATION  #1 | ("COVID-19" OR "COVID 19" OR "COVID-19 Virus Disease" OR "COVID  19 Virus Disease" OR "COVID-19 Virus Diseases" OR "COVID-19 Virus  Infection" OR "COVID 19 Virus Infection" OR "COVID-19 Virus Infections"  OR "2019-nCoV Infection" OR "2019 nCoV Infection" OR "2019-nCoV  Infections" OR "Coronavirus Disease-19" OR "Coronavirus Disease 19" OR  "2019 Novel Coronavirus Disease" OR "2019 Novel Coronavirus Infection"  OR "2019-nCoV Disease" OR "2019 nCoV Disease" OR "2019-nCoV  Diseases" OR "COVID19" OR "SARS Coronavirus 2 Infection" OR "SARSCoV-2 Infection" OR "SARS CoV 2 Infection" OR "SARS-CoV-2 Infections"  OR "COVID-19 Pandemic" OR "COVID 19 Pandemic" OR " COVID-19  Pandemics" OR "SARS-CoV-2" OR "Coronavirus Disease 2019 Virus" OR  "2019 Novel Coronaviruses" OR "Wuhan Seafood Market Pneumonia  Virus" OR "SARS-CoV-2 Virus" OR "SARS CoV 2 Virus" OR "SARS-CoV-2  Viruses" OR "Wuhan Coronavirus" OR "SARS Coronavirus 2" OR  "coronavirus disease 2019" OR "2019 novel coronavirus epidemic" OR  "coronavirus disease 2" OR "coronavirus disease 2010" OR "coronavirus  disease 2019 pneumonia" OR "coronavirus infection 2019" OR " COVID"  OR "COVID 19 induced pneumonia" OR "COVID 2019" OR "COVID-10" OR  "COVID-19 induced pneumonia" OR "COVID-19 pneumonia" OR "nCoV  2019 disease" OR "nCoV 2019 infection" OR "new coronavirus  pneumonia" OR "novel coronavirus 2019 disease" OR "novel coronavirus  2019 infection" OR "novel coronavirus disease 2019" OR "novel  coronavirus infected pneumonia" OR "novel coronavirus infection 2019"  OR "novel coronavirus pneumonia" OR "paucisymptomatic coronavirus  disease 2019" OR "SARS coronavirus 2 pneumonia" OR "SARS-CoV-2  disease" OR "SARS-CoV-2 pneumonia" OR "SARS-CoV2 disease" OR  "SARS-CoV2 infection" OR "SARSCoV2 disease" OR "SARSCoV2 infection"  OR "severe acute respiratory syndrome 2" OR "severe acute respiratory  syndrome 2 pneumonia" OR "severe acute respiratory syndrome  coronavirus 2 infection" OR "severe acute respiratory syndrome  coronavirus 2019 infection" OR "severe acute respiratory syndrome CoV-  2 infection" OR "Wuhan coronavirus disease" OR "Wuhan coronavirus  infection" OR "Severe acute respiratory syndrome coronavirus 2" OR  "2019 nCOV" OR "2019 new coronavirus" OR "2019 novel coronavirus"  OR "2019 severe acute respiratory syndrome coronavirus 2" OR "2019-  nCoV" OR "COVID 19 virus" OR "HCoV-19" OR "Human coronavirus 2019"  OR "nCoV-2019" OR "novel coronavirus 2019" OR "novel coronavirus-19"  OR "SARS-related coronavirus 2" OR "Sever acute respiratory syndrome  coronavirus 2" OR "Severe acute respiratory coronavirus 2" OR "severe  acute respiratory syndrome 2 virus" OR "severe acute respiratory  syndrome corona virus 2" OR "severe acute respiratory syndrome4  coronavirus 2019" OR "severe acute respiratory syndrome CoV-2 virus"  OR "Severe acute respiratory syndrome related coronavirus 2" OR "Severe  acute respiratory syndrome virus 2") |
| CONCEPT  #2 | (Telemedicine OR "Mobile Health" OR mHealth OR eHealth OR "digital health" OR teleconsultation OR "long distance consultation" OR "teleconsultation" OR "telephone consultation" OR electronic consultation OR  "e-consultation" OR econsultation OR "remote consultation" OR  teleconsultations OR telehealth OR "e-health" OR "tele-health" OR  telediagnosis OR "remote diagnoses" OR "remote diagnosis" OR "remote  diagnostics" OR "tele-diagnosis" OR telediagnoses OR telediagnostics OR  telemonitoring OR "distant monitoring" OR "distant patient monitoring"  OR "remote monitoring" OR "remote patient monitoring" OR "tele  monitoring" OR "video consultation" OR "telemedicine videoconsultation" OR videoconsultation OR telepharmacy OR "telepharmacy") |
| CONTEXT  #3 | ("Primary Health Care" OR "Primary Healthcare" OR "Primary Care" OR  "first line care" OR "primary care nursing" OR "primary healthcare" OR  "primary nursing care" OR "primary medical care" OR "primary care") |

| **SEARCH STRATEGY FOR GRAY LITERATURE** | |
| --- | --- |
| #4 | ("COVID-19" OR "SARS-CoV-2") AND (Telemedicine OR teleconsultation OR  "digital health" OR "electronic consultation" OR "remote consultation" OR telehealth) AND ("Primary Health Care" OR "Primary Care" OR "primary medical care") |

| **DATA SOURCE** | **QUERY** | **RESULTS** |
| --- | --- | --- |
| MEDLINE/PUBMED | #1 AND #2 AND #3  filters: none | 416 |
| SCOPUS |  | 480 |
| WEB OF SCIENCE |  | 243 |
| CINAHL |  | 196 |
| EMBASE |  | 300 |
| LILACS |  | 57 |
|  | | |
| GOOGLE SCHOLAR | #4  filters: none | First 100 results |
| WHO GLOBAL RESEARCH ON CORONAVIRUS DISEASE |  | 317 |
| OPAS TECHNICAL DOCUMENTS AND RESEARCH EVIDENCE ON COVID-19 |  | 9 |
| COCHRANE LIBRARY |  | 10 |
| MEDRVIX |  | 40 |
| SCIELO PREPRINTS |  | 5 |
| PREPRINTS.ORG |  | 6 |
| OPEN GREY |  | 0 |
| GREY LITERATURE REPORT (GREYLIT) |  | 0 |
