# Supplementary material for: Digital Health Opportunities to Improve Primary Health Care in the Context of COVID-19: Scoping Review
Source: JMIR Hum Factors. 2022 May 31;9(2):e35380. doi: 10.2196/35380 (PMC9159467; doi:10.2196/35380)
Supplement: Multimedia Appendix 3 [file humanfactors_v9i2e35380_app3.doc]

# Multimedia Appendix 3: Stakeholders consultation guide

After reading the main results of the article "Digital health strategies and their impacts on quality of care in Primary Health Care in the context of COVID-19: A Scoping Review", reply to the following questions:

1. In the scientific field, the dissemination of academic research results are very important to academia, services, management and the entire community. Therefore, we would like your suggestion on forms of dissemination of the results of this study.
2. How do you think this study could have applicability to primary health care services?
3. From this scoping review, what ideas would you give for future studies?
